# *Candida auris*: Epidemiology and Antifungal Strategy

**DOI:** 10.1146/annurev-med-061523-021233

**Published:** 2025-01-16

**Authors:** Emily F. Eix, Jeniel E. Nett

**Affiliations:** Department of Medicine and Department of Medical Microbiology and Immunology, University of Wisconsin–Madison, Madison, Wisconsin, USA

**Keywords:** *Candida auris*, epidemiology, treatment, drug resistance, transmission, antifungal, microbiome, skin colonization

## Abstract

*Candida auris* is a recently emerged fungal pathogen that causes severe infections in healthcare settings around the globe. A feature that distinguishes *C. auris* from other fungal pathogens is its high capacity to colonize skin, leading to widespread outbreaks in healthcare facilities via patient-to-patient transmission. *C. auris* can persist on skin or in the surrounding environment for extended periods of time, and it exhibits greater antifungal resistance than other *Candida* species. These factors pose major obstacles for the prevention and treatment of *C. auris* infection. Recent reports have identified frequently colonized skin sites, risk factors for developing invasive infection, and patterns of antifungal resistance among *C. auris* strains, all of which help guide therapeutic options. In this review, we highlight key studies of *C. auris* epidemiology and antifungal resistance, discussing how these factors influence healthcare-associated transmission and treatment outcomes.

## EPIDEMIOLOGY

### Emergence

*Candida auris* was first described in 2009 after it was isolated from the ear canal of a hospitalized patient in Japan (1). By 2013, *C. auris* had been identified in India, South Korea, South Africa, and Venezuela, and it has since spread globally (2–6). Whole-genome sequencing of 47 isolates has identified at least four distinct clades corresponding with geographic regions: South Asia (clade I), East Asia (clade II), South Africa (clade III), and South America (clade IV) (7). The clades differ significantly, but within clades isolates display high genetic similarity. These results suggest that *C. auris* did not originate from a single source but rather emerged simultaneously in different regions (7). In 2022, a fifth clade was identified in Iran, separated from the other clades by more than 200,000 single-nucleotide polymorphisms (8).

The United States reported its first isolates in 2016 (9). The majority of these isolates (90%) clustered to the South Asian clade, with 7% clustering to the South American clade and one isolate each belonging to the South African and East Asian clades (10). These findings suggest multiple introductions of *C. auris* to the United States during this time, mostly through documented travel from international healthcare systems with ongoing outbreaks (10). Similar findings from outbreaks in the United Kingdom point to numerous introductions of *C. auris* (11, 12). Understanding the clades and species involved in outbreaks is particularly relevant for *C. auris*, as isolates from the clades differ in traits such as biofilm growth, drug resistance patterns, and pathogenicity (13). Interestingly, isolates from the East Asian clade are generally not associated with invasive infection and are less resistant to antifungals (13, 14).

### Skin Colonization and Nosocomial Transmission

In medical facilities, *C. auris* persists on high-touch surfaces, medical devices, and patients’ skin (12, 15, 16). This high propensity for skin colonization enables the fungus to spread rapidly among patients. *C. auris* sheds from skin and persists on medical equipment or surfaces, creating a reservoir in the environment of hospitals or other long-term care facilities (12, 17). As *C. auris* persists for weeks in the hospital environment, these reservoirs become sources of continuous transmission (18). Further complicating this issue is the inability of commonly used antiseptics, such as chlorhexidine gluconate, to eradicate *C. auris* from skin, even with daily treatments (17, 19). Although *C. auris* exhibits susceptibility to chlorhexidine in vitro, this susceptibility does not appear to translate to use on skin, possibly because of limited skin penetration or practical issues with administration (20–22). Clinical studies have shown that, due to the difficulty of decolonizing *C. auris* from skin, *C. auris* can persist on skin for months, and models predict that patients may remain colonized for more than a year (16, 23).

Long-term skin colonization contributes greatly to the high transmissibility of *C. auris* among patients (23). During *C. auris* outbreaks, the most commonly sampled sites include the nares, axilla, and groin, likely due to adoption of screening protocols for the skin colonizer and pathogen methicillin-resistant *Staphylococcus aureus* (24). As these skin sites typically have high burdens of *C. auris*, they remain recommended sampling locations. However, other body sites may also house *C. auris* and contribute to spread. For example, in a study attempting to identify all colonized patients, a skilled nursing facility implemented screening of additional body sites (23), recovering *C. auris* from the hands, toe webs, and perianal skin. Patients negative for *C. auris* at the commonly tested body sites (nares, axilla, groin) were often positive at other body sites; ~70% of residents were colonized at two or more body sites (23). This study suggests that colonization of skin sites that are not routinely screened may contribute to prolonged transmission in healthcare facilities.

Until recently, *C. auris* was frequently misidentified via standard phenotypic identification techniques. For example, many Colombian clinical isolates initially classified as *Candida haemulonii* were later identified as *C. auris* with the use of matrix-assisted laser desorption/ionization–time of flight mass spectrometry and DNA-sequencing tests (25, 26). Retrospective analyses of fungemia cases in South Korea revealed three instances in which *C. auris* bloodstream isolates were misidentified as *C. haemulonii* (3). One of these strains had been isolated from a patient in 1996, suggesting that *C. auris* existed prior to its initial identification in 2009 but had not been broadly circulating. Inaccurate identification can result in treatment delays, so it is critical that *C. auris* be quickly and correctly identified to prevent unnecessary spread.

### Risk Factors, Clinical Infection, and Current Burden

**Figure 1:**
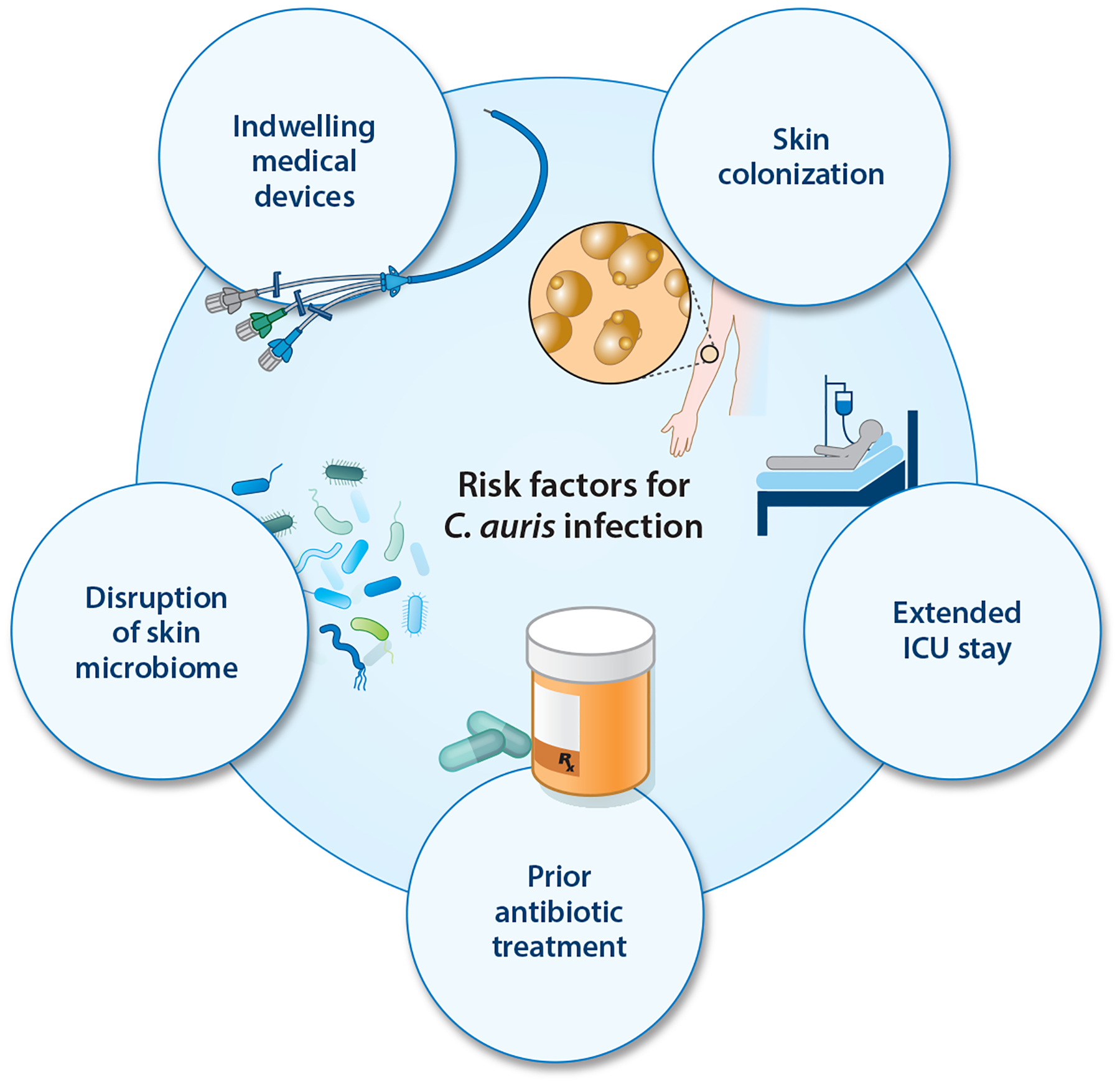
Risk factors for hospital-associated *Candida auris* infection. Abbreviation: ICU, intensive care unit.

Most patients colonized with *C. auris* do not develop invasive disease, but when bloodstream infection occurs, mortality rates are alarmingly high (7, 12, 16). Common risk factors for the development of invasive disease include the insertion of catheters and other medical devices, prior antibiotic treatment, extended stays in an intensive care unit, prior surgeries, and other underlying medical conditions (7, 16, 27–30) (Figure 1). In a Pakistani hospital outbreak, 97% of *C. auris* patients had an indwelling vascular line, which was implicated as the primary source of candidemia for 89% of those with *C. auris*, in contrast to only 46% of patients with candidemia due to other species (30). The same study found that 37% of the *C. auris* candidemia patients had received prior antifungal treatments versus only 1% of those with non–*C. auris* candidemia (30). These results highlight the distinct patterns of *C. auris* infection in comparison to other species.

Reported mortality rates for patients with *C. auris* candidemia typically range from 30% to 60% (2, 7, 31–35). A study by the Centers for Disease Control and Prevention (CDC) that analyzed 192 *C. auris* cases in a US hospital database cites a crude mortality rate of 34% (35). This study identified several underlying conditions common among *C. auris* patients: sepsis, diabetes, chronic kidney disease, and pneumonia. These comorbidities are associated with *C. auris* infections across numerous studies and likely contribute to the wide range of mortality rates observed for *C. auris* patients (2, 32, 34). Notably, some *C. auris* clades appear to be more commonly associated with bloodstream infection than others. A wide-scale analysis of multiple *C. auris* outbreaks showed that clade I and IV isolates have a higher rate of bloodstream infection than clade II or III (33). This finding correlates with evidence of clade-specific differences in phenotypic traits and emphasizes the importance of clade identification during outbreaks.

Recent studies have shed light on the role of *C. auris* in the skin microbiome. *Malassezia* represents the primary fungal genus on the skin of healthy adults, whereas *Candida* species are rarely present (36). However, among patients in long-term care facilities, skin microbiome samples reveal a high prevalence of *Candida* species, especially in facilities experiencing *C. auris* outbreaks (23, 36, 37). Skin microbiomes dominated by *Malassezia* species appear generally more resistant to *C. auris* colonization, but once *C. auris* colonizes skin it quickly predominates (23). The presence of *C. auris* also alters the bacterial community in the microbiome, shifting it toward a predominance of more potentially pathogenic Proteobacteria species (36). These studies suggest that dysbiosis of the skin microbiome may represent another risk factor for *C. auris* colonization, and further studies of *C. auris* within the microbiome are an interesting area for future research.

### Environmental Reservoirs

It is hypothesized that *C. auris* existed in the environment before emerging as a human pathogen (38). Since its documented human spread, *C. auris* has been isolated from marine environments, including a salt marsh wetland devoid of human activity (39). Compared with other *Candida* species, *C. auris* exhibits high salt and temperature tolerance, likely promoting its growth in such environments. These traits are speculated to have arisen from the effects of climate change, and potentially independently for each of the clades (38). While the origin of *C. auris* and the implication of its existence in the environment are as yet unproven, several studies have identified environmental reservoirs for *C. auris* that are relevant for disease tracking. *C. auris* can survive in seawater and river water for up to a month, and it has a strong propensity for biofilm growth on plastic or glass residing in water (40). Surveys of wastewater in Florida and Nevada have detected *C. auris*, with higher abundances measured in wastewater coming from healthcare facilities (41, 42). This type of environmental survey may be useful as a public health tool for early detection of *C. auris* outbreaks.

## ANTIFUNGAL TREATMENT

### Scope of Drug Resistance

In comparison to other *Candida* species, *C. auris* isolates exhibit higher resistance to available antifungals (7, 33, 43, 44). The CDC currently lists tentative minimum inhibitory concentration (MIC) breakpoints (in micrograms per milliliter) as fluconazole ≥32, amphotericin B ≥2, anidulafungin ≥4, caspofungin ≥2, and micafungin ≥4 (45). A recent analysis of 67 studies containing data from 33 countries with more than 4,000 *C. auris* cases revealed extraordinarily high rates of fluconazole resistance (91%), with lower resistances to amphotericin B (12%), caspofungin (12%), micafungin (0.8%), and anidulafungin (1.1%) (33). While MICs appear higher for caspofungin than for micafungin, caspofungin is a more potent inducer of paradoxical growth at concentrations above the MIC (Eagle effect), perhaps complicating MIC interpretation (46). However, this paradoxical growth does not appear to correlate with in vivo outcomes, and higher MICs may not necessarily link to poor patient outcomes (46).

While fluconazole resistance is uniformly found among isolates across geographic regions, MICs to echinocandins and amphotericin B vary (33, 43, 44). For example, within the United States, amphotericin B resistance is reported to be below 5% in the Midwest and West and 85% in the Mid-Atlantic (43). As *C. auris* disease is caused by circulating strains, understanding resistance patterns can inform decision-making at the local level. For instance, a study from Qatar reported 85% resistance to amphotericin B, highlighting the importance of initiating echinocandins as first-line therapy for this outbreak (44). While echinocandins are recommended as first-line therapy for most patients, MICs vary by geography and outbreak. A multicountry analysis found that isolates from India have a higher rate of caspofungin resistance (23.6%) than isolates from other countries (0.2%) (33). Echinocandin resistance remains low in the United States but has occasionally emerged for patients on therapy, and these echinocandin-resistant strains are now presumed to be circulating at low levels (43). While the data for monomicrobial resistance are concerning, the identification of pan-resistant isolates exhibiting resistance to all available drug classes (echinocandins, azoles, and amphotericin B formulations) is even more alarming (47, 48).

### Therapeutic Options

Three antifungal drug classes have been approved for the treatment of invasive candidiasis: echinocandins (e.g., caspofungin, micafungin, anidulafungin), triazoles (e.g., fluconazole, voriconazole), and amphotericin B formulations (49) (Table 1). Given the high resistance rates for *C. auris*, MIC testing [Clinical and Laboratory Standards Institute (CLSI) or European Committee on Antimicrobial Susceptibility Testing (EUCAST)] is routinely recommended for isolates from patients requiring treatment (45). Treatment should be reserved for patients with a documented invasive infection and avoided for cases of colonization at nonsterile sites, such as the skin or respiratory tract (45). In light of their more favorable susceptibility profiles, echinocandins are recommended as first-line therapies for most patients and should be initiated pending susceptibility testing (45, 50, 51). Notably, pharmacodynamic modeling studies in mice have shown that echinocandins exhibit potent activity against *C. auris*; the pharmacodynamic target for micafungin associated with fungal stasis is ≥20-fold lower than targets for other *Candida* species (52). This finding suggests that *C. auris* isolates with higher MICs may be successfully treated with echinocandins beyond what has been achieved for other *Candida* species with similarly high MICs. Additionally, mounting evidence shows that echinocandins, particularly micafungin, may be safely dosed in adults above the typical 100 mg daily regimen (53). Therefore, higher dosing of micafungin may be a consideration for the treatment of *C. auris* isolates with elevated echinocandin MICs in the setting of limited treatment options.

Some limitations for echinocandin therapy include parenteral-only formations, lack of accessibility to these agents in some resource-limited areas, and reduced penetration to the central nervous system (CNS) and urine (54) (Table 1). Echinocandins also lack penetration to the eye and should not be relied upon for the treatment of vitritis or endophthalmitis (49). The most commonly prescribed echinocandins (caspofungin, micafungin, anidulafungin) are dosed daily. However, a newer formation (rezafungin) exhibits a longer half-life, allowing for weekly dosing, and it is currently approved as an alternative agent for the treatment of candidemia and invasive candidiasis. Like other echinocandins, rezafungin exhibits activity against *C. auris*, and murine studies suggest that current dosing would achieve the pharmacodynamic targets for more than 90% of *C. auris* isolates (55).

While echinocandins are the preferred agents for *C. auris* treatment, transition to liposomal amphotericin B can be considered for patients who are not clinically responding or have persistent candidemia for more than 5 days (45). *C. auris* isolates exhibit variable MICs to voriconazole and other second-generation triazoles (56). It is currently recommended to use susceptibility to fluconazole as a surrogate for triazole resistance (45). However, the use of voriconazole or other second-generation triazoles can be considered on a case-by-case basis. The role of flucytosine for the treatment of *C. auris* is not clearly defined. Guidelines from the Federation of Infectious Diseases Societies of Southern Africa suggest that combination therapy with flucytosine can be considered for a short period pending results of susceptibility testing (50). For the treatment of invasive candidiasis due to other species, flucytosine is generally administered as part of a combination therapy and reserved for refractory infections, such as endocarditis, meningitis, and endophthalmitis (49).

While most reports of *C. auris* outbreaks include adult patient populations, *C. auris* also causes outbreaks in neonatal intensive care units, resulting in high mortality (25). As neonates are particularly prone to *Candida* meningitis, the recommended first-line agent is amphotericin B in light of its CNS tissue penetration (49). However, this approach may be problematic for *C. auris* treatment because many isolates exhibit high MICs to amphotericin B (57). Therefore, echinocandins have often been employed in this setting (57). Echinocandins can be used in the neonatal population, but they are typically reserved for cases where meningitis has been excluded (45). As meningitis frequently remains a concern for neonates younger than 2 months, amphotericin B deoxycholate is the preferred antifungal for this patient population.

Given the high levels of resistance reported for *C. auris*, there has been interest in assessing the efficacy of dual antifungal therapy. In vitro and modeling studies suggest that some combinations of amphotericin B, triazoles, echinocandins, and flucytosine may have additive or synergistic activity (58–60). Other studies report primarily indifferent interactions without antagonism (61, 62). Routine use of combination therapy is not currently recommended for the treatment of *C. auris*. However, it can be considered on a case-by-case basis for seriously ill patients as well as those with relapsed or persistent candidemia (45, 50). In an outbreak of more than 70 patients, 45% required a second agent due to nonresponse or persistent fungemia while on echinocandin monotherapy (63). Most of these patients received liposomal amphotericin B (63). Combination therapy can also be considered for infections involving the CNS and/or urine, given the limited penetration of echinocandins to these sites (51). In addition, the use of combination therapy and/or amphotericin B monotherapy is recommended for patients who develop *C. auris* candidemia in the setting of echinocandin prophylaxis (64).

### Antifungals in the Pipeline

Ibrexafungerp represents a first-in-class member of the triterpenoids that exhibits activity against fungal glucan synthase. While echinocandins also target β-1,3-glucan synthase, their inhibitory mechanism differs from that of ibrexafungerp, and ibrexafungerp displays activity against some echinocandin-resistant *C. auris* isolates (56). Ibrexafungerp is currently available as an oral formulation and has been approved for the treatment of acute or recurrent vulvovaginitis. It was recently evaluated in an open-label phase III clinical trial designed to assess efficacy and safety for patients with *C. auris* infection (NCT03363841). Ibrexafungerp has not been directly compared with echinocandins for the treatment of *C. auris*. The CDC currently lists ibrexafungerp as a consideration for the treatment of pan-resistant *C. auris* infection (45).

Fosmanogepix is currently under development as another first-in-class antifungal. This prodrug rapidly converts the active form (manogepix) via mammalian phosphatases and impairs fungal growth through inhibition of Gwt1 (the fungal glycosylphosphatidylinositol-anchored wall transfer protein 1) (65). Fosmanogepix exhibits potent activity against *C. auris* and many other *Candida* species, with the notable exception of *C. krusei* (56). Fosmanogepix recently completed a phase II study for the treatment of candidemia caused by *C. auris* (NCT04148287) (66). In this open-label single-arm study, nine study participants received intravenous treatment followed by oral dosing. Treatment success and survival were reported for 89% of patients. Uniformly low MICs were observed for manogepix (0.004–0.015 μg/mL), and no drug-related adverse events were reported. Fosmanogepix will next be assessed for the treatment of candidemia/invasive candidiasis in a phase III study with a comparison to the current standard of care (echinocandin followed by fluconazole) (NCT05421858). Like ibrexafungerp, the CDC lists fosmanogepix as a potential treatment for pan-resistant *C. auris* infection (45).

### Antifungal Duration and Management

For patients with uncomplicated invasive infection and/or candidemia, guidelines recommend an antifungal duration of at least 2 weeks from bloodstream clearance (49–51). Longer therapeutic durations are required for patients with more complicated infections, such as osteomyelitis, meningitis, endocarditis, or endophthalmitis (49).

Surveillance blood cultures not only help guide antifungal duration but also assess for persistent candidemia. Reports of breakthrough fungemia (defined as persistent fungemia after 3 days of antifungal treatment) are common (28%) (14). As resistance can develop while patients are receiving therapy, repeat MIC testing is recommended to evaluate the need for an alternative agent (45, 47, 50). Occurrence may even happen later in therapy. In a retrospective, case–control study of patients with candidemia, Simon et al. (67) found higher microbiologic recurrence at 60 days for patients with *C. auris* (11.9%) versus those with other *Candida* species (4%), but rates of persistent candidemia for both groups were similar.

Central venous catheters are common for patients diagnosed with invasive *C. auris* infection; some studies report the presence of catheters in up to 100% of patients (25, 30, 68, 69). In a study of patients in Kenya, patients with *C. auris* candidemia were more likely to have an indwelling central line preceding fungemia (84%) in comparison to patients with *Candida albicans* candidemia (54%) (70). *C. auris* and other *Candida* species form adherent biofilms on the surfaces of vascular catheters and other foreign material (71). Like biofilms formed by other *Candida* species, *C auris* biofilms are highly tolerant of antifungal therapy, withstanding manyfold-higher concentrations than those needed to kill nonbiofilm *C. auris* (21, 72). In a case series, a mortality rate above 80% was observed for patients without prompt catheter removal (44). Another report described similarly high mortality (84%) for patients without source control (30). However, these observational studies may be confounded by patients with more severe illness who select comfort measures without intrusive medical interventions, such as catheter removal and replacement.

Current guidelines recommend the removal of vascular catheters in the setting of candidemia, as well as consideration for the removal of other devices, including infected urinary catheters or other prosthetic material (49, 50). Similar to the treatment of invasive candidiasis caused by other species, timely drainage of abscesses is recommended for source control (49, 50). Guidelines also recommend dilated eye exams for patients with candidemia. Eye exams appear to be particularly important for patients with *C. auris*, given the high usage of echinocandins and the poor penetration of these compounds into eye compartments (49).

## CONCLUSIONS AND FUTURE DIRECTIONS

*C. auris* is a major public health threat that continues to rise globally. Central to this problem is the capacity of *C. auris* to colonize skin and persist in the hospital environment accompanied by high rates of antifungal resistance. Given the distinct clinical traits of *C. auris*, it is clear that we must find new approaches to treat and mitigate the spread of this deadly pathogen. Studies have shed light on the hallmarks of colonization and clinical disease, the risk factors associated with *C. auris* bloodstream infection, and the growing threat of drug resistance.

## Figures and Tables

**Table 1:** Antifungals for the treatment of *Candida auris*

Antifungal	First line, age ≥2 months	First line, age <2 months	Preferred alternative	Central nervous system/eye	Urine	Notes
Echinocandins	+	−	−	−	−	Lowest reported MICs
Amphotericin B deoxycholate	−	+	−	+	+	Side effects of electrolyte disturbance and kidney injury
Liposomal amphotericin B	−	−	+	+	−	Side effects of electrolyte disturbance and kidney injury
Fluconazole	−	−	−	+	+	High MICs and frequent resistance

Abbreviation: MIC, minimum inhibitory concentration.
